# 

**Published:** 1846-04

**Authors:** 


### BOOKS RECEIVED FOR REVIEW.

1. Ventilation. A Reply to the Misstatements in reference to ships and buildings ventilated by the author. By D. B. Reid, M.D. F.R.S.E. London, 1845. 8vo, pp. 28.

2. Appendix to an Essay on Therapeutical Inquiry. By James Arnott, M.D. Brighton, 1845. 12mo, pp. 35.

3. On the Importance of Chemistry to Medicine. An Introductory Address and Inaugural Lecture. By W. A. Miller, M.D. F.R.S., Professor of Chemistry, King's College. London, 1846. 8vo, pp. 38.

4. Unhealthiness of Towns: its Causes and Remedies. A Lecture. By R. D. Grainger, Esq. London, 1845. Small 8vo, pp. 47.

5. The Potato Disease; its Origin, Nature, and Prevention. By G. Phillips. London, 1845. 8vo, pp. 58. 2s. 6d.

6. Observations on the Nosological Arrangement of the Bengal Medical Returns. By T. J. Mouat, M.D. Calcutta, 1845. 8vo, pp. 64.

7. Report on the Amount and Causes of Death in Manchester. By J. Robertson, Esq. London, 1845. 8vo, pp. 23.

8. Introductory Lecture on the present position of some of the most important of the modern Operations of Surgery By J. D. Mütter, M.D. Philadelphia, 1844. 8vo, pp. 34.

9. Notes of a Microscopical Examination of Chalk and Flint. By. G. A. Mantell, LL.D. F.R.S. London, 1845. 8vo, pp. 24.

10. A Natural History of the Mammalia. By G. R. Waterhouse. Part I. London, 1845.

11. Report on the Health of Exeter. By T. Shapter, M.D. London, 1845. 8vo, pp. 47.

12. Observations on the Mineral Waters of Homburg. By F. H. Prytharch, M.D. Homburg, 1845. 12mo, pp. 88.

13. The Nature and Treatment of Cancer. By W. H. Walshe, M.D. Lond. 1845. 8vo, pp. 590. 16s.

14. An Inaugural Address, delivered at the opening of the Norfolk and Norwich Hospital Museum. By J. S. Crosse, M.D. F.R.S. Norwich, 1845. 8vo, pp. 28. 1s. 6d.

15. An Address, delivered at the opening of the Classes of the Medical School attached to the Middlesex Hospital. By A. Shaw, Esq. London, 1845. 8vo, pp. 32.

16. Die angebornen Verrenkungen. Von L. J. Melicher, M.D. Wien, 1845. 8vo, pp. 220.

17. An Address of the Society of Apothecaries to the General Practitioners of England and Wales. London, 1845. 8vo, pp. 29. 1s.

18. Memoir on Amputation at the Hip-joint. By W. S. Cox, F.R.S., Senior Surgeon of the Queen's Hospital, Birmingham. London, 1845. folio, pp. 47. 16s.

19. A Practical Treatise on Abdominal Hernia. By T. P. Teale, F.L.S., Surgeon to the Leeds General Infirmary. With numerous Illustrations. London, 1846. 8vo, pp. 383. 15s.

20. On Tubercle of the Brain in Children. By J. M. Adams. Glasgow, 1846. 8vo, pp. 32.

21. Abstract of Researches on Magnetism, and on certain allied subjects. By Baron Reichenbach. Translated and abridged by W. Gregory, M.D. F.R.S.E. London, 1846. 8vo, pp. 112.

22. Instructions for making Unfermented Bread. With observations. By a Physician. London, 1846. 8vo, pp. 15. 3d.

23. Remarks on Slavery and Slave Trade in the Brazils. By T. Nelson, Assist. Surg. R.N. London, 1846. 8vo, pp. 85.

24. Cyclopædia of Anatomy and Physiology. Part XXVII. March, 1846. 5s.

25. Mercury in Fevers, Dysentery and Hepatitis, as they occur in India, &c. By J. S. Sutherland, M.D. Edinburgh, 1846. 8vo, pp. 95.

26. Transactions of the Provincial Medical and Surgical Association. Vol. XIV. London, 1846. 8vo, pp. 285. 15s.

27. Chelius's Surgery. Translated by J. F. South. Part. 10. 3s.

28. An Essay on the Treatment of Compound and Complicated Fractures. By W. F. Walker, M.D. Boston (U.S.), 1845. 8vo, pp. 101.

29. On the Human Mouth. By Alex. Nasmyth, Surgeon Dentist to the Queen. London, 1846. 8vo, pp. 23.

30. Lectures, illustrative of various subjects in Pathology and Surgery. By Sir B. C. Brodie, Bart. London, 1846. 8vo, pp. 411. 12s.

31. A System of Practical Surgery. By W. Fergusson. Second Edition. 8vo, pp. 668. 12s. 6d.

32. A Manual of Natural Philosophy. By J. L. Comstock, M.D., and R. D. Hoblyn, A.M. London, 1846. 8vo, pp. 477.

33. Practice of Surgery. By James Miller, Professor of Surgery in the University of Edinburgh. Edinburgh, 1846. 8vo, 9s.

34. Phrenology Examined. By P. Flourens. Translated by C. D. L. Meigs, M.D. Philadelphia, 1846. 12mo, pp. 144.

35. The London Medical Directory, 1846. London, 1846. 8vo, pp. 270. 5s. 6d.

36. Observations and Essays on the Statistics of Insanity, &c. By John Thurman. London, 1845. 8vo, pp. 334. 14s.

37. Lectures on the Nature and Treatment of Deformities. By R. W. Tamplin. London, 1846. 8vo, pp. 267. 7s. 6d.

38. The Hunterian Oration for 1846. By W. Lawrence, F.R.S. London, 1846. 8vo, pp. 68. 2s.

39. Transactions of the Medical Society of London. New Series. Vol. I. London, 1846. 8vo, pp. 221. 9s.

40. Medical Notes on China. By John Wilson, M.D. F.R.S. London, 1846. 8vo, pp. 267. 10s.

41. A Treatise on Scrofula, its Nature, its Causes, its Prevalence and Treatment. By Benjamin Phillips, F.R.S. London, 1846. 8vo, pp. 386.

42. Die Nervenkraft im Sinne, gegenüber dem Blutleben in der Natur. Von Dr. C. J. Heidler. Braunschweig, 1845. 8vo, pp. 392.

43. A Manual of Physiology, including Physiological Anatomy. By W. B. Carpenter, M.D. F.R.S. 8vo, pp. 582.

44. The Half-yearly Abstract of the Medical Sciences. July—Dec. 1845. W. H. Ranking, M.D. 8vo, pp. 472. 6s. 6d.

### INDEX TO VOL. XXI

OF THE

### BRITISH AND FOREIGN MEDICAL REVIEW.

PAGE

ABDOMEN, penetrating wounds of . 421
Abortion, Dr. Meigs on . . 90
Abscess of the liver, Dr. Budd on . 46
Acetic acid in scarlatina . . 497
Adaptation, general, in nature . 112
Adventitious products, development of 418
Allopathy and Homœopathy . 225
Anatomy, elements of . . 499
first steps to . . 493
and physiology, report on 541
Aneurism, operation for . . 287
treatment of, by pressure 490
Apoplexy, meningeal, memoir on 409
passive, in children . 385
Armies, on the formation of . 166
Arnott, Dr. on compression in cancer 220
his inventions . . 218
Arnott, Dr. Jas. on therapeutical inquiry 498
Arteries, ligature of . . 287
Asphyxia, Mr. Erichsen on . . 265
Auscultation, Dr. Hughes on . 281
Badham, Dr. his insect life . 494
Bennet, Dr. on diseases of the uterus 174
Berlin, medical topography of . 205
Bile, derangement of, Dr. Budd on 56
its nature and uses . . 40
Births, deaths, and marriages in England 2
other countries ib.
Bleeding in diseases of the brain . 103
Blood, state of, in marshy districts . 211
Bombay, medical transactions of . 373
Bones and joints, treatises on diseases of 124
Bonnet, M. on diseases of the joints ib.
Books received for review . 284, 570
Bowerbank, Mr. on the structure of shells 62
Brain, hypertrophy of, in children . 386
inflammation of, in children 385
of bleeding in diseases of . 103
softening of, in children . 390
and nervous centres, Dr. Todd on 452
Brain and spinal marrow, diseases of 381

PAGE

Bronchotomy, Mr. Miller on . 488
Brown, Mr. on scarlatina . . 497
Budd, Dr. on diseases of the liver . 39
Cancer of the liver, Dr. Budd on . 59
the nature and treatment of 218
nature and progress of . 219
effect of compression on . 220
case of, by Dr. Walshe . 223
Dr. Walshe's treatment in . 421
nosological position of . 422
chemistry of . . 426
physiology of . . ib.
pathology of . . 428
treatment of . . 432
Carpenter, Dr. on structure of shells 62, 76
his manual of physiology 480
Cartilage, diseases of . . 131
Cases by Dr. Forbes . . 539
Cazenave on diseases of the skin . 491
Cephalhæmatoma . . . 384
Cerebral congestion in children . 382
Chapman, Dr. on eruptive fevers, &c. 358
Cheek, plastic operations on . 305
Chemistry, its rank as a science . 110
Children, on diseases of the brain of 381
Children's hospital at St. Petersburg 470
Chlorosis, treatment of . . 89
Churchill, Mr. his manuals . 479
Cicatrices, operations for . . 289
Cirrhosis of the liver . . 49
Classification of diseases, Schönlein's 27
Clinical medicine, Schönlein's . 33
Clitoris, case of enlarged . . 85
Clubfoot, tenotomy applied to . 182
Colles, Mr. his lectures on surgery . 271
Colloid cancer . . . 424
Colombat, M. his treatise on women 83
Combe, Dr. A. on the observation of nature . . . . 505
Combe, Mr. G. letter to . . 509
Commissioners for towns, report of 333

PAGE
Compression, its effect in cancer . 220
Congenital luxations, treatise on . 393
Congestion of the liver, Dr. Budd on 43
cerebral, in children . 382
Contagion, Schönlein's views of . 21
Convulsions in infancy . . 391
Cowpox, history of, in America . 362
Critical terminations of diseases . 23
Crosse, Dr. his inaugural address . 283
Deafness, Dr. Kitto on . . 188
Delirium, acute, of the insane . 418
Dieffenbach's operative surgery . 285
Digestion, Dr. Prout on . . 109
Diseases, on the origin of . . 145
Disease, natural course of, always interfered with . . . 252
Diseases, the history and treatment of 501
Dr. Laycock on the natural history of . . 525
Doses, infinitesimal, in homœopathy, account of . . . 228
Drainage and sewerage of towns . 333
Dropsy, Dr. Chapman on . . 369
Drummond, Dr. his first steps to anatomy 493
Drysdale and Russel on homœopathy 225
Dublin, sanitary condition of . 1
Duration of life in different classes . 10
Duties and rights of medical men . 438
Ear, plastic operations on . . 302
Encephaloid cancer . . 423
Enteritis, treatment of . . 93
Eruptive diseases, Chapman's lectures on 358
cause of . . . 98
diagnosis of . . 96
Epistaxis, Dr. Chapman on . . 367
Erichsen, Mr. on asphyxia . . 268
Eyelids, plastic operations on . 308
Falconer, Dr. his fossil zoology . 476
Farinacea and fruits the food of man 148
Fauna antiqua Sivalensis . . 476
Fergusson, Mr. his manual of surgery 483
Fever, Schönlein's notion of . 22
Fingers and toes, operations on . 330
Fistula lacrymalis, operations for . 316
Fleischmann, Dr. his report of diseases 241
Flooding, Dr. Meigs on . . 90
Forbes, Dr. his illustrations of mesmerism . . . . 277
Foundling hospital of St. Petersburg 466
Fossil zoology of the north of India . 476
Fractures, accidental, in the drowned 485
Frontal sinus, case of polypus of . 186
Fruits and farinacea the food of man 148
Fuligno, a physician of the 14th century 279
Gall-bladder, inflammation of . 51
Gallstones, Dr. Budd on . . 58
Geigel, M. on the origin of disease . 145
Genoa, medical society of . . 274
Glottis, œdema of . . . 412
Gout, the nature and treatment of . 215

PAGE
Gout, decrease of in America . 371
Griffin, Messrs. their medical problems 92
Guy, Dr. on the duration of peers' life 1
Hahnemann, his works, history, and doctrines . . . 225
Hair, structure and development of 202
Health of towns commission . 333
Hematemesis, Dr. Chapman on . 368
Hemoptysis, Dr. Chapman on . 366
on the treatment of . 107
Hemorrhages, critical . . . 23
Dr. Chapman on . 366
Hemorrhoids, Dr. Chapman on . 368
Homœopathy and allopathy 225-502
Henderson, Dr. on homœopathy . 225
Hereditary transmission of diseases 417
Homœopathy, estimate of doctrines of 234
Hughes, Dr. his manual of auscultation 281
Hydrocephalus, Dr. Mauthner on . 386
Hydrothorax, treatment of . . 370
Hypertrophy of the brain in children 386
Impotence and sterility . . 89
Improvement of health, bill for the . 333
Inflammation of the liver, Dr. Budd on 44-9
of the brain in children 385
Insanity, Dr. Herzog on . . 460
medical jurisprudence of . 416
Jackson, Dr. Robt. his life and writings 166
Jaundice, Dr. Budd on . 55-60
Joints, pathological anatomy of . 125
effects of immobility on . 141
experiments on . 136-143
and bones, various treatises on diseases of . . 124
Julie, Mademoiselle, a curer of diseases 534
Jurisprudence, Mr. Taylor's manual of 484
Kitto, Dr. on deafness . . 188
Lacrymal fistulæ, operations for . 310
Laryngismus stridulus, Dr. Griffin on 104
Laurie, Dr. on homœopathy . 225
Laycock, Dr. on the natural history of disease . . . . 525
Life, animal and vegetable, Dr. Shearman on . . . 477
insect, by Dr. Badham . 494
duration of that of peers . 1
Life-tables, Mr. Neison's . . 5
Limbs, position of in disease of joints 137
Litzmann, Dr. on puerperal fever . 275
Liver, anatomy of . . 39
Dr. Budd on diseases of . ib.
Lunatics, Dr. Winslow on the act for 276
Lungs, disease of in pregnancy . 90
Luxations, congenital, treatise on . 593
Mammalia, natural history of . 496
Mammals, odontography of . 62
Man, his natural food . . 148
Mauthner, Dr. on the diseases of children 381
Manufacture of noses . . 294
Marchetti, Sig. on the Tuscan maremma 205

70

PAGE
Maremma of Tuscany, statistics of . 205
Measles in America . . 364
Medical deontology . . 438
essays of St. Petersburg . 460
profession, proposed new organization of . . 531
Medicines, homœopathic, mode of preparing . . . . 236
Meigs, Dr. his translation of Colombat 83
Melicher, Dr. on congenital luxations 393
Memoirs of Royal Academy of Medicine 408
Menstruation, singular neglect in . 84
Mesmerism, Dr. Forbes on . 277
Meteorology, Dr. Prout's treatise on 109
Middlesex Hospital, history of . 282
Miller, Professor, his surgery . 488
Mortality in England . . 3
Mouth, plastic operations on . 303
Muscles and tendons, operations on 331
Nævi, operations for . . 290
Natural history of the mammalia . 496
disease, mode of obtaining 525
Nature, her power to cure diseases . 253
on the observation of, in disease 505
Neison, Mr. his vital statistics . 1
Nosology, new system of, by Schönlein 27
Nerves, operations on . . 332
Nervous system, on diseases of . 273
Neuroses, spinal, treatise on . 280
Norfolk hospital museum . . 283
Nose, plastic operations on . . 294
Observation of nature in disease . 505
Odontography, Professor Owen's . 62
Œdema of the glottis, memoir on . 412
Operations, rhinoplastic . . 294
Operative surgery, Dieffenbach's . 285
Ophthalmology, Rognetta's treatise on 158
Opium, inquiry into its therapeutic effects 105
Osteitis, observations on . . 128
Ovaria, diseases of . . . 88
Owen, Professor, his odontography . 62
Paget, Mr. his report on anatomy and physiology . . . 541
Palate, operations on . . 306
Parker, Mr. Langston, on syphilis . 495
Patronage of quacks and impostors . 533
Peerage, duration of life amongst . 1
Perineum, ruptured, operations for . 323
Pessary, Dr. Meigs on the use of . 86
Physiology, Dr. Carpenter's manual of 479
and anatomy, report on 541
Plastic surgery, Dieffenbach on . 292
Polypus of the frontal sinus, case of . 186
Pregnancy, extra-uterine, case of . 419
Prepuce, manufacture of . . 313
Prisoners, on the weight of . . 278
Problems, medical, Messrs. Griffin's 92
Prolapsus uteri, operation for . 326
Prout, Dr. his treatise on meteorology 109
Prurigo vulvæ, treatment of . 87

PAGE
Puberty, epoch of . . . 84
Puerperal fever, Dr. Meigs on . 92
treatise on . . 275
Quain, Dr. his anatomy . . 499
Recto-vaginal fistulæ, operations for 322
Rectum, operations on . . 328
Reformation of medicine, suggestions for the . . . . 262
Registrar-General, his annual report 1
Rhumatism, acute, treatment of . 108
treatment of, by nature 372
Robertson, Dr. on the nature and treatment of gout . . . 215
Rognetta, M. on ophthalmology . 158
Sanitary condition of Dublin . 1
Scarlatina, Dr. Chapman on . 364
treatment of . . 497
Schönlein, his pathology and therapeutics 20
his natural system of diseases 25
Scirrhus (cancer) . . . 424
Scrotum, operations on . . 312
Sebastian, M. his elements of physiology 282
Senses, the lost—deafness . . 188
Sewerage and drainage of towns . 333
Sharpey, Dr. his Quain's anatomy . 599
Shearman, Dr. on animal and vegetable life . . . . . 473
Shells, on the structure of . . 62
Simon, M. on medical deontology . 438
Skin, healthy, Mr. Wilson's treatise on 198
M. Cazenave, on diseases of . 491
diseases, classification of . 492
Smallpox, Dr. Chapman on . 358
Smith, Mr. on the food of man . 148
Softening of the brain in children . 390
Speculum, on the use of . . 175
Spinal irritation, case of . . 99
remarks on . 101
neuroses, treatise on . 280
Statistics, vital, contributions to . 1
St. Petersburg, medical essays of . 460
Stricture of the rectum, treatment of 329
Suicides in England in 1840 . 3
Surgery, Mr. Colles's lectures on . 271
operative, Dieffenbach's . 285
Mr. Fergusson's manual of 483
Professor Miller's . 488
Synovitis, M. Bonnet on . . 127
Symptoms of disease, Schönlein on . 21
Syphilis, modern treatment of . 495
Taylor, Mr. his manual of jurisprudence 481
Teeth, comparative anatomy of . 62
structure of . . 70
Tendons and muscles, operations on 331
Tenotomy applied to clubfoot . 182
Therapeutics, general, Schönlein on . 24
Dr. J. Arnott on . 498
Todd, Dr. his anatomy of the brain, &c. 452
Toes and fingers, operations on . 330
Topography, medical, of Berlin . 205

PAGE
Towns, large, sanitary state of . 333
Trachea, operations on . . 312
Transactions of medical society, Bombay 373
Tumours, operations on . . 290
Typhus abdominalis, Schönlein on 34
Urethra, plastic operations on . 313
Urethroplasty, M. Segalas on . 408
Urine, secretion of, its relation to poisoning . . . . 420
Urinary infiltrations in lithotomy . 489
Uteri cervix, inflammation and ulceration of . . . 179
Uterus, abscess of . . . 85
 polypus and prolapsus of . 86
 various diseases of . . 87
 treatise on diseases of neck of 174
 operations on . . 326
Vagina, anatomy of . . 84
 malformations of . . 85
 plastic operations on 315-321
Valleix, M. on œdema of the glottis 412

PAGE
Varicose tumours, operations for . 288
Vesico-vaginal fistulæ, operations for 315
Vienna, homœopathic hospital of . 240
Walshe, Dr. his treatise on cancer 218-421
Waterhouse, Mr. on the mammalia . 496
Weight of prisoners . . 278
Weis, Dr. on clubfoot and tenotomy 182
Willis, Mr. on the sanitary condition of Dublin . . . 1
Wilson, Mr. his treatise on the skin 198
 his history of Middlesex hospital 282
Winslow, Dr. on the lunatic act . 276
Wollheim, M. on the topography of Berlin . . . 205
Wood, Dr., his homœopathy unmasked 225
Woods, influence of, on health . 209
Women, treatise on diseases of . 83
Wounds of the abdomen, penetrating 421
Wounds of the head, insanity from . 464
Wuth, Dr. his contributions to medicine 186
Zoology, fossil, of India . . 476

END OF VOL. XXI.

C. AND J. ADLARD, PRINTERS, BARTHOLOMEW CLOSE.

